# Corrigendum

**DOI:** 10.1111/iwj.14363

**Published:** 2023-08-29

Corrigendum to “Sub epidermal moisture measurement and targeted SSKIN bundle interventions, a winning combination for the treatment of early pressure ulcer development” (Int Wound J. 2022 Dec 27. doi: 10.1111/iwj.14061)

Byrne S, Patton D, Avsar P, et al. Sub epidermal moisture measurement and targeted SSKIN bundle interventions, a winning combination for the treatment of early pressure ulcer development. Int Wound J. 2023;20 (6):1987–1999. doi:10.1111/iwj.14061

In Section 3.7.2, the percentage reduction value reported in the original article was incorrect. The text currently reads: The OR of SEM PU ≥0.5 was 0.59 (95% CI: 0.24–1.00; *p* = 0.05), indicating a 49% reduction in the odds of SEMPU ≥0.5 in the treatment group and this finding is statistically significant. This should read: The OR of SEM PU ≥0.5 was 0.59 (95% CI: 0.24–1.00; *p* = 0.05), indicating a 41% reduction in the odds of SEMPU ≥0.5 in the treatment group and this finding is statistically significant.

In the Conclusion section, the threshold value for sub‐epidermal moisture (SEM) delta was incorrectly reported as 0.05. The correct threshold value for the SEM delta is ≥0.5.

We apologize for this error.

